# Quality of Life of Children with Cystic Periventricular Leukomalacia – A Prospective Analysis with the Child Health Questionnaire-Parent Form 50

**DOI:** 10.3389/fped.2016.00050

**Published:** 2016-05-17

**Authors:** Bernhard Resch, Anja Mühlanger, Ute Maurer-Fellbaum, Elisabeth Pichler-Stachl, Elisabeth Resch, Berndt Urlesberger

**Affiliations:** ^1^Research Unit for Neonatal Infectious Diseases and Epidemiology, Medical University of Graz, Graz, Austria; ^2^Division of Neonatology, Department of Pediatrics, Medical University of Graz, Graz, Austria; ^3^Outpatient Department of Developmental Follow-Up, Department of Pediatrics and Adolescent Medicine, Medical University of Graz, Graz, Austria

**Keywords:** cerebral palsy, Child Health Questionnaire, periventricular leukomalacia, preterm infant, quality of life

## Abstract

**Objective:**

Cystic periventricular leukomalacia (PVL) is associated with moderate to severe physical and mental handicaps in preterm infants. We hypothesized whether or not those handicaps were associated with a poorer quality of life (QOL) of affected children and their families compared to matched controls.

**Patients and methods:**

All children with the diagnosis PVL collected from a local database of the Division of Neonatology of the Medical University of Graz, Austria, and born between 1997 and 2008 were included in the study group. Preterm infants matched for gestational age, birth weight, year of birth, and gender without PVL served as controls. Selected perinatal data and neurological outcome were documented. The interview of the parents was conducted using the Child Health Questionnaire-Parent Form 50 (CHQ-PF50), German version. The CHQ-PF50 consists of 50 items divided over 11 multi-item scales and 2 single-item questions.

**Results:**

The CHQ-PF50 was answered by 21 parents of the study (26%) and 44 of the control (39%) group. Cases were diagnosed as having developmental delay, dystonia, strabismus, central visual impairment, seizures, and cerebral palsy (81 vs. 7%, *p* < 0.001) more common than controls. Analysis of the CHQ-PF 50 revealed significantly poorer results for cases regarding physical health (physical functioning: *p* < 0.001, physical social limitations: *p* < 0.001, and physical summary score: *p* < 0.001). Several psychosocial categories (behavior, mental health, and self-esteem) and the psychosocial summary score did not differ between groups. Only two categories (parental impact concerning time *p* = 0.004 and family activities: *p* = 0.026) revealed significantly poorer results in the cases as it was for the global category for health (*p* = 0.009).

**Conclusion:**

Children with PVL had an overall poorer QOL regarding physical aspects. However, PVL was not generally associated with a poorer QOL regarding psychosocial aspects.

## Introduction

Children with cerebral palsy may experience various degrees of limited mobility, self-care capability, restrictions in communication, and restrictions in participation, which threaten their health-related quality of life (HRQOL). The concept of HRQOL aims to measure the subjective impact of a condition and its treatment upon the patient’s everyday life and health experience, and thus is inherently subjective (1). The quality of life (QOL) as defined as a person’s perception of their well-being and satisfaction with life is a multidimensional construct that includes elements about general functioning, as well as the person’s appraisal of their life experiences and social/emotional well-being (2). Use of generic QOL measures provides information that is not specific to the disease process and enables one to compare perceived QOL with universal values (3). Results of a study including 59 children at a mean age of 9 years with almost half of them having mild motor impairment (47% Gross Motor Function Classification System level I) indicated that QOL was highly variable in children with cerebral palsy, with about half of them experiencing a life quality similar to typically developing children, and motor and other activity limitations were indicators of physical but not psychosocial well-being (2). Additionally, family functioning, behavioral difficulties, and motivation were important predictors of social/emotional adaptation. The authors concluded that determinants of life quality might guide resource allocation and health promotion initiatives to optimize health of the child and family (2). Maternal psychological distress, coping, parenting/marital stress, child health, and family impact were measured in very low birth weight (VLBW) children at the age of 8 years showing that mothers of VLBW children differed from term mothers, reporting less consensus with partners, more concern for their children’s health, less parent–child conflict, and fewer years of education attained (4). Mothers of high-risk VLBW children even experienced the greatest family and personal strains and used less denial and disengagement coping implicating that VLBW birth has long-term negative and positive impacts on maternal/family outcomes related to the infant’s medical risk.

The Child Health Questionnaire (CHQ) is one of the most widely used tools for assessing health status in children and as a generic instrument for measuring health outcomes, well-being, and functional status, the CHQ has been used worldwide in studies evaluating children with a wide range of diagnoses (5). The CHQ has been demonstrated to be valid and reliable in many settings with an internal consistency median reliability coefficient of 0.84; thus, the CHQ includes questions aimed at assessing a wide range of domains applicable to a broad spectrum of children (5). Cystic periventricular leukomalacia (PVL) is rarely associated with normal development, implicates mental retardation in half of the cases, cerebral palsy in more than 80%, visual impairment in nearly a quarter of cases, rarely hearing impairment, and seizure disorders in a quarter of affected children (6). No study explicitly investigated QOL in this population using the Child Health Questionnaire-Parent Form 50 (CHQ-PF50).

We hypothesized whether or not those handicaps were associated with a poorer subjective QOL of the affected children and their families by conducting a case–control study using preterm infants without diagnosis of PVL as matched controls.

## Patients and Methods

In this CHQ study, children with diagnosis of cystic PVL grades 2–4 according to the description by de Vries et al. (7) hospitalized at the Division of Neonatology of the Medical University of Graz, Austria, a tertiary care referral hospital, and born between 1997 and 2008 were available for inclusion. Following approval of the local ethic committee (Number 25-384 ex 12/13) data from these children were collected from a large local database published recently (6). Controls included children without diagnosis of PVL matched for gestational age (±1 week), birth weight (±100 g), gender, and year of birth. The medical charts and the data from our outpatient clinic of neurodevelopmental follow-up were reviewed.

Parents were contacted by phone and informed about the study (Anja Mühlanger), thereafter they received the CHQ-PF50 by post including a stamped envelope to send back the filled out questionnaire.

The interview of the parents was conducted with the CHQ-PF50, German Version (8). It was completed, as recommended, by each child’s parent or guardian, who was instructed to answer the 50 items with the child in mind (5). The CHQ, consisting of 50 items, was scored using the customary algorithm, which calculates subscale scores ranging from 0 to 100, with higher scores indicating better health status (9–11). Any written comments about the CHQ items provided by the respondents were recorded. The CHQ form was not altered or amended in any way, and the respondents were given no additional guidance while completing the form. The CHQ assesses for 13 physical and psychosocial domains: general health perceptions, physical functioning, role/social physical functioning, self-esteem, mental health, general behavior, bodily pain, role/social emotional/behavioral functioning, parent impact – time, parent impact – emotional, family activities, family cohesion, and at least change in health over the last year (10, 11). Answers have to be given covering the last 4 weeks of their child, except change in health that covers the last year of the child and is calculated between 5 (now much better) and 1 (now much worse). General health and behavior each consist of 1 question calculating 0 points for being bad, 30 points for less good, 60 points for good, 85 points for very good, and 100 points for excellent (9). An overview of the CHQ-PF50 is given in Table 1 according to Raat et al. (11).

**Table 1:** **CHQ-PF50: scales, items per scale, and score interpretation (11)**.

Scale	Number of items	Description low score	Description high score
Physical functioning (PF)	6	Child is limited a lot in performing all physical activities, including self-care, due to health	Child performs all types of physical activities, including the most vigorous, without limitations due to health
Role functioning: emotional/behavior (REB)	3	Child is limited a lot in school work or activities with friends as a result of emotional or behavior problems	Child has no limitations in schoolwork or activities with friends as a result of emotional or behavior problems
Role functioning: physical (RF)	2	Child is limited a lot in school work or activities with friends as a result of physical health	Child has no limitations in schoolwork or activities with friends as a result of physical health
Bodily pain (BP)	2	Child has extremely severe, frequent and limiting bodily pain	Child has no pain or limitations due to pain
General behavior (BE)	6	Child very often exhibits aggressive, immature, delinquent behavior	Child never exhibits aggressive, immature, delinquent behavior
Mental health (MH)	5	Child has feelings of anxiety and depression all of the time	Child feels peaceful, happy, and calm all of the time
Self-esteem (SE)	6	Child is very dissatisfied with abilities, looks, family/peer relationships, and life overall	Child is very satisfied with abilities, looks, family/peer relationships, and life overall
General health perceptions (GH)	6	Parent believes child’s health is poor and likely to get worse	Parent believes child’s health is excellent and will continue to be so
Parental impact: emotional (PE)	3	Parent experiences a great deal of emotional worry/concern as a result of child’s physical and/or psychosocial health	Parent does not experience feelings of emotional worry/concern as a result of child’s physical and/or psychosocial health
Parental impact: time (PT)	3	Parent experiences a lot of limitations in time available for personal needs due to child’s physical and/or psychosocial health	Parent does not experience limitations in time available for personal needs due to child’s physical and/or psychosocial health
Family activities (FA)	6	The child’s health very often limits and interrupts family activities or is a source of family tension	The child’s health never limits or interrupts family activities nor is a source of family tension
Family cohesion (FC)	1	Family’s ability to get along is rated “poor”	Family’s ability to get along is rated “excellent”
Change in health (CH)	1	Child’s health is much worse now than 1 year ago	Child’s health is much better now than 1 year ago

Statistical analyses were done with *t*-test and Wilcoxon test for numerical data after checking the normality assumption with the Kolmogorov–Smirnov test. Categorical data were tested with chi-square using Yates correction and Fisher’s exact test as appropriate. Analysis was done with SPSS version 17 (SPSS Inc., 2008, Chicago, IL, USA) and Microsoft Excel 2007 (Microsoft Corporation, 2007, Redmond, WA, USA). A *p*-value <0.05 was considered to be significant.

## Results

**Figure 1:**
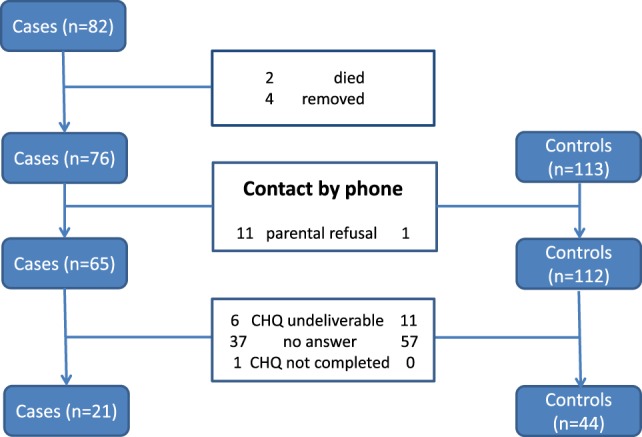
**Flow chart representing the complete study cohort of 82 preterm infants diagnosed as having cystic PVL and 113 matched controls without PVL born between 1997 and 2008 and the final results of completed CHQ**. PVL, periventricular leukomalacia; CHQ, Child Health Questionnaire.

From 82 cases available for inclusion during the study time period 21 (26%) sent back a valid CHQ-PF50 that was compared to 44 (39%) questionnaires from 113 matched controls (see flow chart diagram in Figure 1). Perinatal data shown in Table 2 proved adequate matching of groups.

**Table 2:** **Perinatal data of 21 cases with diagnosis of cystic periventricular leukomalacia (PVL) and 44 matched controls without PVL born between 1997 and 2008**.

	Cases	Controls	*p*-Value
Age (years)	11	11	0.740
Male sex	11 (52)	27 (61)	0.495
Gestational age (weeks)	30	31	0.099
Birth weight (g)	1470	1492	0.799
Multiple birth	6 (29)	17 (38)	0.458
Cesarean section	12 (57)	37 (84)	0.026
Apgar at 1 min	6.7	6.9	0.229
Apgar at 5 min	8.7	8.7	0.309
Apgar at 10 min	9.1	9.1	0.610
Mechanical ventilation	17 (84)	32 (73)	0.351

*Data are given as *n* (%) or median values*.

Neurodevelopmental outcome differed significantly between groups (see Table 3). More children in the case group had diagnosis of cerebral palsy (81 vs. 7%, *p* < 0.001) and less had diagnosed as having normal cognitive development (29 vs. 64%, *p* < 0.001).

**Table 3:** **Neurodevelopmental outcome of 21 cases with diagnosis of cystic periventricular leukomalacia (PVL) and 44 matched controls without PVL born between 1997 and 2008**.

	Cases	Controls	*p*-Value
Age at follow-up (months)	73 (17–138)	45 (12–91)	0.006
Normal cognitive development	6 (29)	28 (64)	<0.001
Developmental delay	11 (52)	8 (18)	<0.001
Mental retardation	4 (19)	6 (14)	0.61
Minimal cerebral dysfunction	4 (19)	0 (0)	0.021
Cerebral palsy	17 (81)	3 (7)	<0.001
Diplegia	5 (30)	3 (100)	0.075
Hemiplegia	4 (23)	0 (0)	0.012
Tetraplegia	8 (47)	0 (0)	<0.001
Strabismus	9 (43)	1 (2)	<0.001
Visual impairment	5 (24)	1 (2)	0.013
Hearing impairment	0 (0)	0 (0)	1.000
Seizures	5 (24)	1 (2)	0.013

*Data are given as *n* (%) or median (range)*.

**Figure 2:**
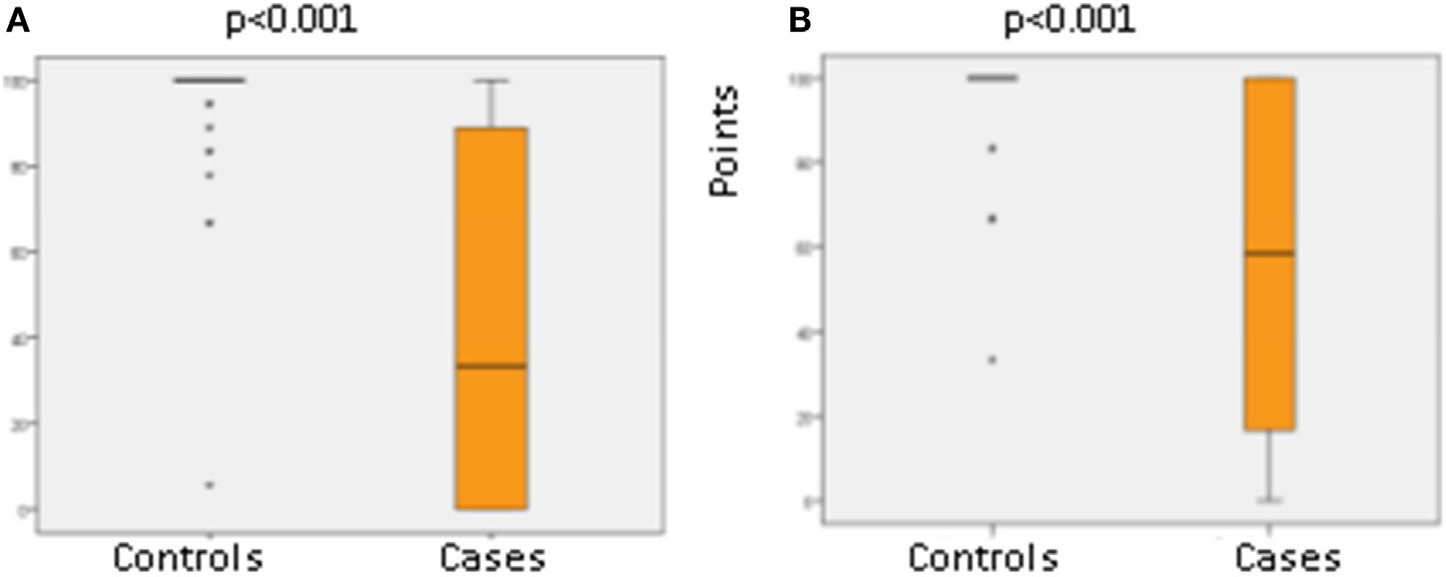
**Physical functioning (A) and role/social physical functioning (B) of 21 cases with diagnosis of cystic periventricular leukomalacia (PVL) and 44 matched controls without PVL born between 1997 and 2008**.

Results of the CHQ-PF50 are shown in Table 4. Significant worse results in the cases were observed regarding general health, physical functioning, role/social physical functioning, parent impact time, and family activities. Analysis of the CHQ-PF50 revealed significantly poorer results for the cases regarding categories that describe the physical health (physical functioning: *p* < 0.001, physical social limitations: *p* < 0.001, and the physical summary score: *p* < 0.001, see Figure 2). Several psychosocial categories (behavior, mental health, and self-esteem) and the psychosocial summary score did not differ between groups besides the two following categories with poorer parental impact concerning time (*p* = 0.004) and family activities (*p* = 0.026) (see Table 4). The global category for health was demonstrated to be significantly poorer in the cases (*p* = 0.009).

**Table 4:** **Results of the Childs Health Questionnaire-parents form 50 comparing 21 children with diagnosis of PVL to 44 without PVL at the median age of 11 years**.

Item of the CHQ-PF50	Cases	Controls	*p*-Value
Physical functioning	45 (43)	95 (16)	<0.001
Role functioning: emotional/behavior	68 (41)	89 (20)	0.077
Role functioning: physical	56 (41)	94 (14)	<0.001
Bodily pain	79 (20)	84 (19)	0.300
General behavior	80 (22)	83 (17)	0.749
Mental health	81 (9)	80 (14)	0.972
Self-esteem	86 (13)	82 (19)	0.924
General health perception	60 (22)	73 (19)	0.070
Parent impact: emotional	58 (30)	72 (24)	0.056
Parent impact: time	56 (35)	81 (22)	0.004
Family activities	68 (27)	84 (20)	0.026
Family cohesion	84 (15)	83 (15)	0.742
Change in health	3.5 (0.8)	3.2 (0.7)	0.670

*Data are given as median (SD)*.

## Discussion

This is so far the first study evaluating QOL in children with diagnosis of PVL using the CHQ-PF50. Not surprisingly, our results demonstrated that PVL was associated with a poorer QOL regarding physical aspects. However, PVL was not generally associated with a poorer QOL regarding psychosocial aspects compared to preterm infants without having the diagnosis of PVL.

Limitations of our study findings, first, include the low response rate in the group of children with PVL that diminishes generalization of study findings for the entire group of affected children. On the other hand, our significant findings are representative according to the CHQ-PF50 manual providing tables that demonstrate small scores necessary to differ from normal values, e.g., reflecting the 68, 90, and 95 percentile (9). Additionally, the manual represents the number need to take part in the questionnaire to find scores differing by 2, 5, 10, or 20 points. All categories with significant differences regarding our study results were found to range without the above provided limits (9). Second, we do not know the factors that pushed families to take or not take part in the study. Active refusal occurred significantly more often in the study group (see Figure 2). The differences regarding rates of cesarean section between groups might not have influenced outcome parameters according to a recent Cochrane review (12). Strength of the study includes the long follow-up time of median far beyond 24 months revealing high-quality neurodevelopmental outcome data for both groups.

Evaluation of the psychometric performance of the CHQ-PF50 including 818 parents of children with cerebral palsy, aged 8–12 years from nine regions of Europe, revealed a large number of ceiling effects for a number of subscales and summary scores across all Gross Motor Function Classification System levels and to a lesser extent floor effects in the physical functioning scale (13). In general, ceiling effects were more commonly observed in psychosocial categories, whereas floor effects were found in physical categories (14). These effects might have influence on the results of the CHQ in severely handicapped children and lessen a further differentiation of physical activities despite suspected higher variance. A further problem in the interpretation of results of the CHQ in children with cerebral palsy is the missing differentiation between disease and handicap that might lead to misinterpretation by the parents (5). Overall, the CHQ provides a good to excellent validity in children with cerebral palsy for physical health more than psychosocial behavior (5, 13). Comparisons with European, Australian, and US studies revealed a satisfying internal consistency and only minor divergences to our study findings (15–18).

Results of the CHQ in preterm infants without diagnosis of PVL were somewhat remarkable regarding all categories that did not differ significantly between groups. We did not expect that several psychosocial categories (behavior, mental health, and self-esteem) and the psychosocial summary score did not differ between groups.

Comparison of results of the CHQ-PF50 of our study group with children diagnosed as having attention deficit hyperactivity disorder, epilepsy, and juvenile rheumatoid arthritis – provided by the CHQ manual (9) – revealed poorer QOL regarding physical aspects but better QOL regarding mental health and self-esteem. Results of social emotional and behavioral functioning were comparable to children with attention deficit hyperactivity disorder, and results of general behavior and health were comparable to children with juvenile rheumatoid arthritis (9).

The psychometric properties of the German translation of the CHQ as used in our study were evaluated in two German clinical samples (19). Item internal consistency (item-scale correlation) and internal consistency of scales were tested; quartiles and factor analysis were conducted and the results of the German clinical samples were compared with US clinical samples. The psychometric testing of the CHQ showed good results with internal consistency of the hypothesized scales being all higher than 0.70.

In conclusion, cystic PVL leads to poorer QOL regarding physical function, but not for psychosocial QOL. Surprisingly, preterm infants with cystic PVL were not found to have significantly worse psychosocial functioning in all areas as compared to control preterm infants.

## Author Contributions

BR was responsible for study design, writing the manuscript, and final approval of the manuscript. AM was responsible for collecting study patients, extracting data from the questionnaire and statistical analysis, and writing tables and figures. ER was responsible for collecting all data of the study cases, and UM-F for providing neurodevelopmental outcome data of all study participants. EP-S participated in study design and questionnaire selection and data analysis. BU was involved in data analysis and interpretation of results. All authors approved the final version of the manuscript.

## Conflict of Interest Statement

The authors declare that the research was conducted in the absence of any commercial or financial relationships that could be construed as a potential conflict of interest.

## References

[1] Mueller-Godeffroy E Thyen U Bullinger M. Health-related quality of life in children and adolescents with cerebral palsy: a secondary analysis of the DISABKIDS Questionnaire in the field-study cerebral palsy subgroup. Neuropediatrics (2016) 47:97–106. 10.1055/s-0036-1571801 26878168

[2] Majnemer A Shevell M Rosenbaum P Law M Poulin C. Determinants of life quality in school-age children with cerebral palsy. J Pediatr (2007) 151:470–5. 10.1016/j.jpeds.2007.04.014 17961687

[3] Guyatt GH Naylor CD Juniper E Heyland DK Jaeschke R Cook DJ Users’ guides to the medical literature. XII. How to use articles about health-related quality of life. JAMA (1997) 277:1232–7. 10.1001/jama.1997.03540390062037 9103349

[4] Singer LT Fulton S Kirchner HL Eisengart S Lewis B Short E Parenting very low birth weight children at school age: maternal stress and coping. J Pediatr (2007) 151:463–9. 10.1016/j.jpeds.2007.04.012 17961686 PMC10228568

[5] Vargus-Adams JN. Inconsistencies with physical functioning and the Child Health Questionnaire in children with cerebral palsy. J Pediatr (2008) 153:199–202. 10.1016/j.jpeds.2008.02.029 18534226

[6] Resch B Resch E Maurer-Fellbaum U Pichler-Stachl E Riccabona M Hofer N The whole spectrum of cystic periventricular leukomalacia of the preterm infant: results from a large consecutive case series. Childs Nerv Syst (2015) 31:1527–32. 10.1007/s00381-015-2786-3 26099229

[7] de Vries LS Eken P Dubowitz LM. The spectrum of leukomalacia using cranial ultrasound. Behav Brain Res (1992) 49:1–6. 10.1016/S0166-4328(05)80189-5 1388792

[8] HealthActCHQ. Child Health Questionnaire-Parent Form 50 CHQ-PF50 German (Austria) Version. Cambridge, MA: HealthActCHQ (2006). Available from: www.healthactchq.com

[9] HealthActCHQ. The CHQ Scoring and Interpretation Manual. Cambridge, MA: HealthActCHQ (2008).

[10] Hullmann SE Ryan JL Ramsey RR Chaney JM Mullins LL Measures of general pediatric quality of life: Child Health Questionnaire (CHQ), DISABKIDS chronic generic measure (DCGM), KINDL-R, pediatric quality of life inventory (PedsQL) 4.0 generic core scales, and Quality of My Life Questionnaire (QoML). Arthritis Care Res (Hoboken) (2011) 63(Suppl 11):S420–30. 10.1002/acr.20637 22588762

[11] Raat H Bonsel GJ Essink-Bot ML Landgraf JM Gemke RJ. Reliability and validity of comprehensive health status measures in children: the Child Health Questionnaire in relation to the health utilities index. J Clin Epidemiol (2002) 55:67–76. 10.1016/S0895-4356(01)00411-5 11781124

[12] Alfirevic Z Milan SJ Livio S. Caesarean section versus vaginal delivery for preterm birth in singletons. Cochrane Database Syst Rev (2013) 9:CD000078. 10.1002/14651858.CD000078.pub3 24030708 PMC7052739

[13] McCullough N Parkes J White-Koning M Beckung E Colver A. Reliability and validity of the Child Health Questionnaire PF-50 for European children with cerebral palsy. J Pediatr Psychol (2009) 34:41–50. 10.1093/jpepsy/jsn048 18499739

[14] McCullough N Parkes J. Use of the Child Health Questionnaire in children with cerebral palsy: a systematic review and evaluation of the psychometric properties. J Pediatr Psychol (2008) 33:80–90. 10.1093/jpepsy/jsm070 17728302

[15] Waters E Salmon L Wake M. The parent-form Child Health Questionnaire in Australia: comparison of reliability, validity, structure, and norms. J Pediatr Psychol (2000) 25:381–91. 10.1093/jpepsy/25.6.381 10980043

[16] McCarthy ML Silberstein CE Atkins EA Harryman SE Sponseller PD Hadley-Miller NA. Comparing reliability and validity of pediatric instruments for measuring health and well-being of children with spastic cerebral palsy. Dev Med Child Neurol (2002) 44:468–76. 10.1111/j.1469-8749.2002.tb00308.x 12162384

[17] Wake M Salmon L Reddihough D. Health status of Australian children with mild to severe cerebral palsy: cross-sectional survey using the Child Health Questionnaire. Dev Med Child Neurol (2003) 45:194–9. 10.1017/S0012162203000379 12613777

[18] Beckung E White-Koning M Marcelli M McManus V Michelsen S Parkes J Health status of children with cerebral palsy living in Europe: a multi-centre study. Child Care Health Dev (2008) 34:806–14. 10.1111/j.1365-2214.2008.00877.x 18959578

[19] Warschburger P Landgraf JM Petermann F Freidel K. Health-related quality of life in children assessed by their parents: evaluation of the psychometric properties of the CHQ-PF50 in two German clinical samples. Qual Life Res (2003) 12:291–301. 10.1023/A:1023233308653 12769142

